# Laws of the State of New York of Interest to Physicians

**Published:** 1914-11

**Authors:** Thomas Carmody


### Laws of the State of New York of Interest to Physicians

The so-called Boylan Bill of the last session of the Legislature became chapter 363 of the Laws of 1914, which amends the Public Health Law in relation to the sale of habit-forming drugs, has given rise to some discussion in this state. Some provisions of the law have been the subject of consideration by the New York State Board of Pharmacy, physicians and others. Attorney-General Thomas Carmody, was called on to render an opinion in relation to the same, which is the official interpretation of those portions of the law under consideration, until otherwise interpreted by some Court of competent jurisdiction. This opinion is of such importance to the medical profession as well as to pharmacists, that it warrants publication in full in the Buffalo Medical Journal, as it has not yet appeared in official series of State Publications. It is as follows:

### Sale of Habit Forming Drugs—Names Required on Prescription and on Retailer's Certificate of Sale.—Public Health Law, Section 246.

Retailers of habit-forming drugs must on sale thereof place on the prescription to be kept on file the name of the physical purchaser, that is, the person to whom the drugs are delivered, not the name of the person for whose use the drug is intended. And the same name also must be written on the label or certificate of sale delivered with the drug.

INQUIRY

From the State Board of Pharmacy I have received a request to construe certain clauses of Chapter 363 of the Laws of 1914, "An Act to amend the public health law, in relation to the sale of habit-forming drugs." Does the statute in requiring the person who sells such drugs at retail to inscribe upon the prescription which he retains on file the "name and address of the purchaser making such purchase," mean that the seller shall place on the prescription the name of the person to whom the drug is actually delivered, or does it mean he shall place thereon the name of the person for whose use the article is intended. Further, does the "name of the person to whom the sale is made," required to be written on the label, intend the name of the patient or the name of the person who presents the prescription and to whom the drug is handed out.

OPINION.

The above statute of 1914, which is an amendment to the Public Health Law, provides at much length as follows concerning the sale of chloral, opium or any of its salts, alkeloids or derivatives or any compound or preparation of any of them:

"§ 246. **Prescriptions; certificates.** It shall be unlawful for any person to sell at retail or give away any of the drugs, their salts, derivatives or preparations mentioned in section two hundred and forty-five of this chapter except as herein provided without first receiving a written prescription signed by a duly licensed physician, veterinarian or dentist. The prescription must contain substantially the following: The name in full of the physician, veterinarian or dentist issuing such prescription, his office address, his office hours, and telephone, and the **name, age, and address of the person to whom, and date on which, such prescription is issued.** It shall be unlawful for any duly licensed physician, veterinarian or dentist **to issue** any such prescription containing any of the drugs, their salts, derivatives or preparations mentioned in section two hundred and forty-five of this chapter **except after a physical examination of any person for the treatment of disease, injury or deformity.** It shall be unlawful for any person to sell at retail any of the drugs or preparations of any of those mentioned in section two hundred and forty-five of this article without first verifying the authority of any prescription containing more than four grains of morphine, thirty grains of opium, two grains of heroin, six grains of codeine or four drams of chloral. Such verification can be made by telephone or otherwise. Such prescription so received shall be filled out at the time of receiving the same for the full quantity prescribed and no prescription so received shall be filled out more than ten days after the date which said prescription be dated. Such prescription, from which no copy shall be taken, shall be retained by the person who dispenses the same and shall be filled but once. Such prescription shall be kept on the general prescription file and given a regular consecutive number on such file. **On such prescription shall be inscribed the name and address of the purchaser making such purchase and the date upon which said sale is made.** Any person who sells at retail, furnishes or dispenses any of the drugs mentioned in section two hundred and forty-five of this chapter upon a written prescription by a duly registered physician or veterinarian or dentist shall at the time of dispensing the same, **place upon the package a label or deliver therewith a certificate stating the name and address of the person selling or furnishing the same, the name and address of the physician, veterinarian or dentist upon whose prescription such sale is made, the date of sale, and the name of the person to whom such sale is made.** Any person other than a manufacturer of any of the drugs mentioned in Section two hundred and forty-five or a wholesale dealer in drugs or a licensed pharmacist, licensed druggist, duly registered practicing physician, licensed veterinarian or a licensed dentist, who shall possess any of the drugs mentioned in section two hundred and forty-five or their salts, derivatives or preparations, shall be guilty of a misdemeanor, unless said possession is authorized by the certificate described in this section.''

Attending first to that portion of the statute which requires the retailer to place upon the prescription the name and address of the purchaser making the purchase, I conclude such direction refers to the name of the actual purchaser, that is, the person who receives the drug from the hands of the retailer, and briefly for these reasons: The physician himself, by earlier provision of the statute, must write upon the prescription blank his office address, his office hours, his telephone number and ''the name, age, and address of the person to whom, and date on which, such prescription is issued.'' A prescription is, I think, in the contemplation of the statute, issued to the patient, not to the person receiving from the physician the paper upon which it is written, in the sense that it runs or pertains to the patient exclusively, such as a written license, though handed to a third person, issues in fact to the person for whom it is intended. This view of the meaning of the word is fortified by the presence of another requirement, that of detailed recital by the physician as to the name, age, and address of the person to whom the prescription is issued, obviously as a means to an opportunity for public officers to investigate, if desired, the necessity for the prescribed drug; and by the use of the word ''issue'' in the next following sentence of the statute, making it unlawful to issue a prescription except after a physical examination of the person.

Having concluded that the physician must place the name of the patient on the prescription, I am easily led to decide that the statute, where it reads ''on such prescription shall be inscribed the name and address of the purchaser making such purchase and the date upon which such sale is made,'' did not intend that the retailer should place the patient's name again upon the paper, but rather, as an added protective or detective measure, the name of the person receiving and taking the drugs away.

On the label or in a separate certificate to be delivered at the time of sale every retailer when dispensing these drugs must place his name and address, the physician's name and address, the date of the sale, and ''the name of the person to whom such sale is made.''

Consistently with my determination that the person receiving the drug from the retailer is the purchaser, ''the person to whom the sale is made'' must likewise be the actual physical purchaser. Some may conceive and urge a benefit in construing the phrase to mean the patient, for his name would then remain upon the package or bottle as a warning that the contents were not for others. The difficulty with that construction is that the statute does not look to the accidental use but to the habitual use of the drug, and it permits the whole statement which the retailer must make out, to appear if the retailer wishes not on the bottle or package but on an entirely separate certificate. Possession of the drugs unless ''authorized by the certificate'' is a misdemeanor. Therefore possession is what the statute seeks to follow, and accordingly the purchaser's name, for he is the first possessor after the retailer, must appear on the certificate.

THOMAS CARMODY,
Attorney-General.

Dated, July 6th, 1914.
To New York State Board of Pharmacy, Albany.

